# Intramedullary dermoid cyst with relatively atypical symptoms: a case report and review of the literature

**DOI:** 10.1186/1752-1947-7-104

**Published:** 2013-04-16

**Authors:** Maryam Sanaullah, Sidra Mumtaz, Akhtar Amin Memon, Abdul Sattar Mohammad Hashim, Sanaullah Bashir

**Affiliations:** 1 Dow Medical College, Dow University of Health Sciences, Baba-e-Urdu Road, Karachi, 74200, Pakistan; 2 Department of Neurosurgery, Dow Medical College, Dow University of Health Sciences, Baba-e-Urdu Road, Karachi, 74200, Pakistan; 3 Jinnah Postgraduate Medical Center, Rafique Shaheed Road, Karachi 35, Pakistan; 4 Civil Hospital Karachi, Dow University of Health Sciences, Baba-e-Urdu Road, Karachi, 74200, Pakistan

**Keywords:** Intermedullary, Dermoid cyst, Conus medullaris, Space-occupying lesion, Spinal tumors

## Abstract

**Background:**

Intraspinal dermoid cysts are rare and benign tumors that occur primarily due to the defective closure of the neural tube, an ectodermal derivative, during the process of development. They are slow-growing tumors manifesting in the second and third decades of life.

**Case presentation:**

We present here a case of a 14-year-old Sindhi boy with a six-month history of paraparesis of the lower limbs and a progressive loss of power of grade 3/5, and hypoesthesia in the L4/L5 dermatomes of his right lower limb. A plain magnetic resonance imaging scan revealed a well-demarcated intraspinal intramedullary cyst containing an abscess at the level of T12 and L1 causing localized cord compression, which was producing the symptoms. Near total excision of the cyst was successfully performed and was sent for biopsy, which revealed keratinocytes and keratin flakes. With one month of follow-up, along with physiotherapeutic management, the patient gradually improved and was able to walk without support.

**Conclusions:**

Critical evaluation of every case with aggravating symptoms should be carried out, and neurological and radiological examinations should be conducted to ensure the well-being of patients.

## Introduction

Amid the numerous defects and deviations from normal development patterns, very few exist as uncommon in medical history as the dermoid cyst. There is no exact knowledge of the prevalence of intracranial dermoid cysts, however, the few studies reported to date propose it to be 0.3 percent [1]. Only a few cases have been reported in the literature [2-4]. Patients with these cysts usually have an extended history of symptoms, on average about 8.5 years between the appearance of symptoms and diagnosis [5,6].

Dermoid cysts can appear subdurally, extramedullary or intramedullary, with intramedullary presentations being relatively rare [7]. Presented here is a case of an intradural intramedullary, space-occupying cyst at the conus medullaris level in a 14-year-old boy together with the magnetic resonance imaging (MRI) and histopathological findings and relevant scientific literature review.

## Case presentation

A 14-year-old Sindhi boy presented to our hospital complaining of having had lower back pain for six months. The pain was followed by the development of weakness and numbness in his lower limbs. He had been constipated for a week prior to reporting. There was no history of urinary incontinence, infection, lumbar puncture, spinal trauma or previous spinal surgery.

The patient was initially diagnosed by a district registered medical officer (RMO) as having tuberculosis (TB) of the spine, the disease being endemic in the area, and was started on antituberculous therapy (ATT). In spite of three months of treatment, the weakness progressed until he became completely bedbound and unable to move. It was then that the patient was moved to a tertiary care hospital.

The patient’s higher mental function was normal and his cranial nerves were intact. An assessment of his motor system revealed a reduced bulk of the flexor group of both lower limbs. There was hypotonia on the left side and power was grade 3/5 in both his lower limbs. Knee and ankle jerks of both lower limbs were absent. The motor examination of the upper limbs was unremarkable with the exception of areflexia, observed bilaterally. On sensory examination, there was hypoesthesia and decreased light touch and pinprick sensation in the L5/L4 dermatomes of his right lower limb. The sacral dermatomal sensations at S1 and S2 levels were also impaired. The overlying skin of the thoracolumber region was intact, showing no signs of inflammation, swelling or hairy nevi, or local hair growth. The rest of the systemic examination was unremarkable. There was no history of weight loss, night sweats, body aches or malaise. Baseline investigations revealed anemia and leukocytosis.

On radiological investigation, an MRI scan of the dorsal spine was performed with T1- (TR/TE 410/19) and T2- (TR/TE 3700/134) weighted images. The MRI scan evidenced a focal, well-defined space-occupying lesion measuring 1.50×0.6cm^2^, and abnormal signal intensity within the spinal canal, which was intradural as well as intramedullary in nature, since there was a splitting of the cord into two layers along the superior aspect of the lesion. The lesion was on the posterior aspect of the cord compressing the conus medullaris at the D12 and L1 level. No perilesional edema was observed.

**Figure 1:**
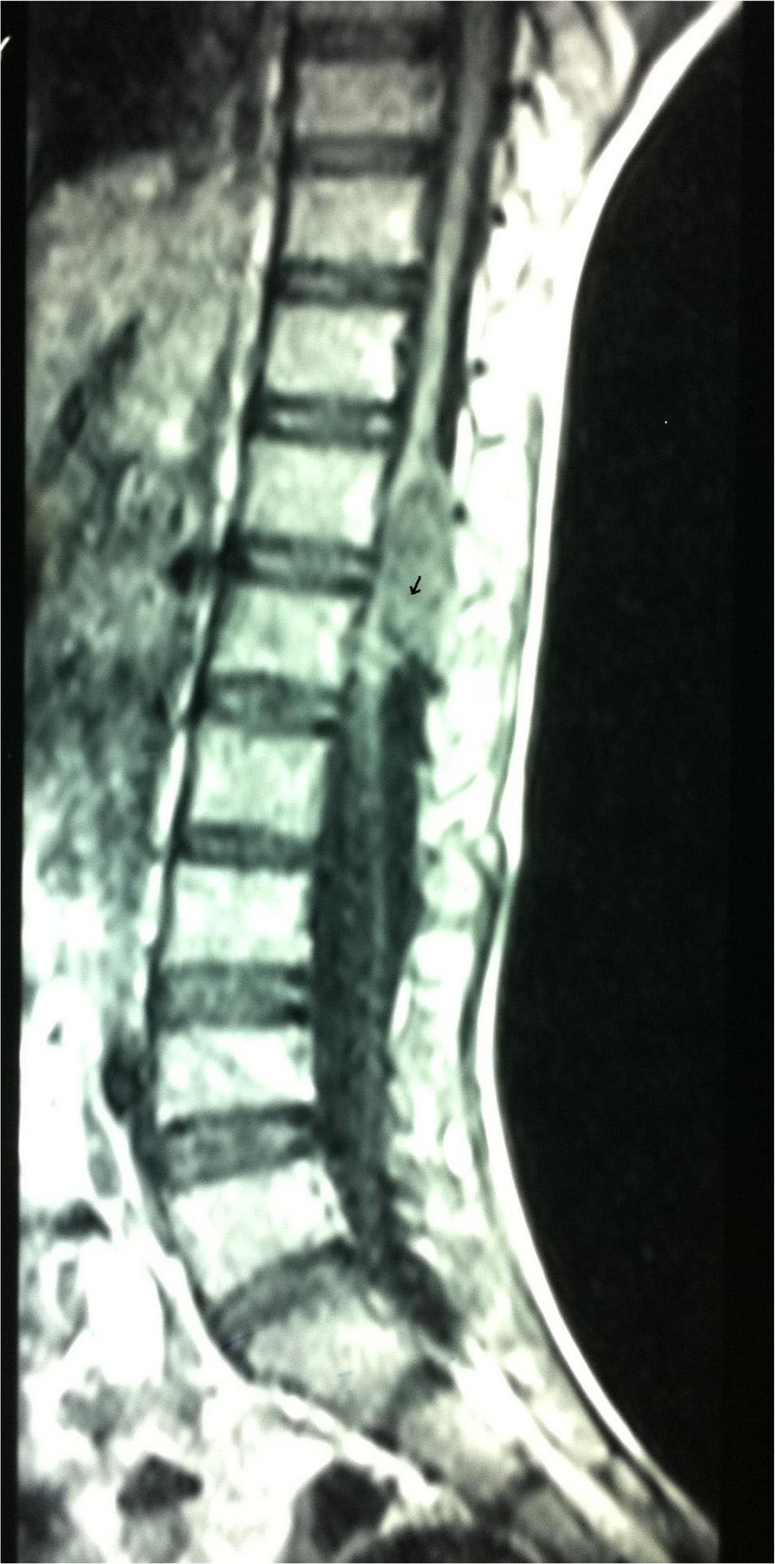
Sagittal T1-weighted image shows a hypointense lesion with a hyperintense signal area at the lower end (arrowhead).

**Figure 2:**
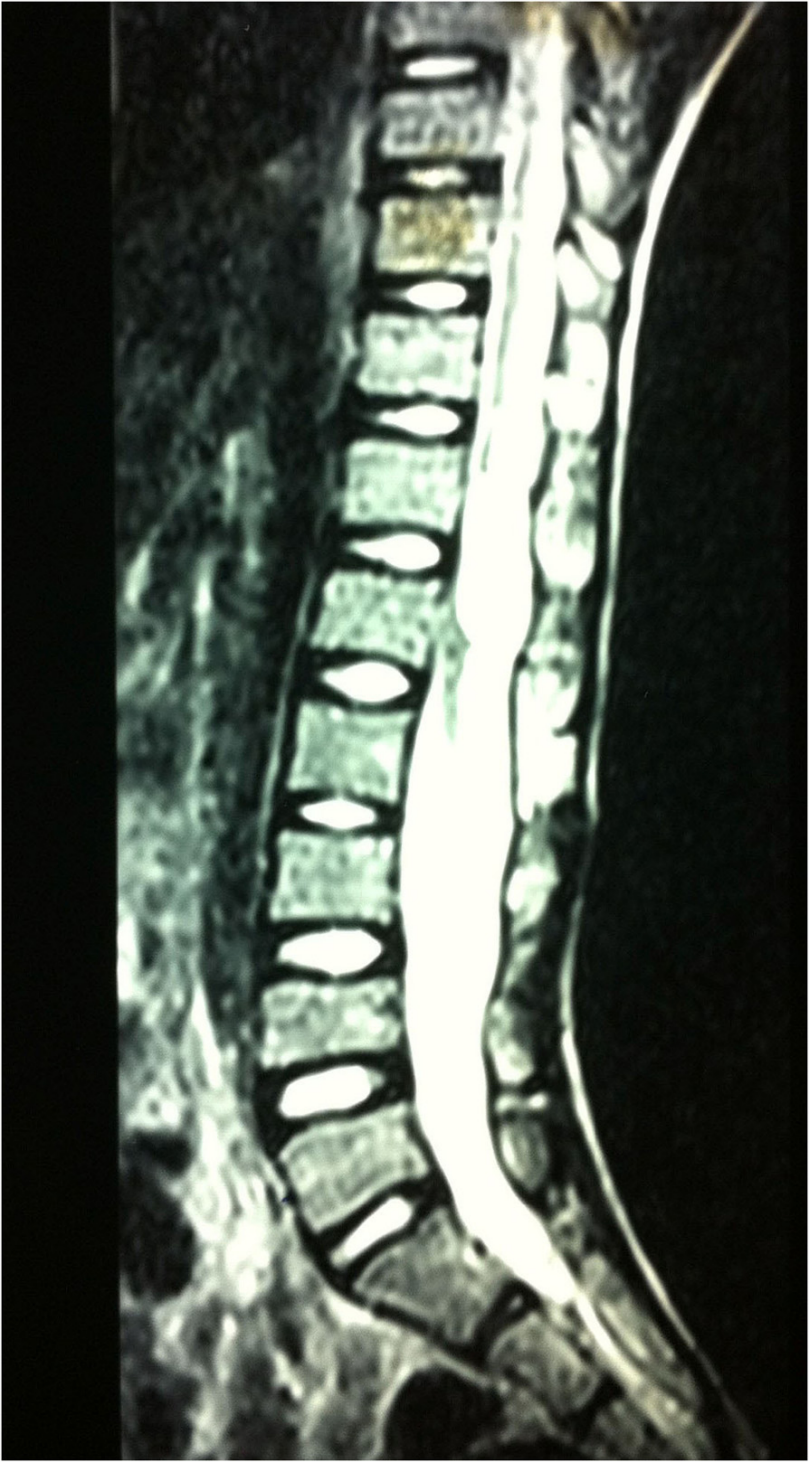
Sagittal T2-weighted axial image showing a homogenously hyperintense lesion.

The tumor was heterogeneous in intensity, localized, and its boundaries were well demarcated. It was hypointense at the T1-weighted frequency with a hyperintense signal area at the caudal end (arrow) (Figure 1) and appeared homogenously hyperintense on the T2-weighted images (Figure 2).

After all aseptic measures, surgical resection was accomplished through a posterior midline linear incision at the D12 and L1 level. All the layers of skin, the subcutaneous fat and the muscles were separated, the dura was opened and a laminectomy was performed. During the operation, the spinal cord was seen to be compressed and overdistended due to an intrinsic lesion. Myelotomy was performed and a well-demarcated, unilocular, grayish white intradural, intramedullary cyst was found involving the conus medullaris and extending from D12 and L1.

The cyst was evacuated and near-total excision was done from the neuronal tissue. Some 0.5ml of thick grayish yellow pus was obtained and sent for histopathology, which revealed aggregates of keratinocytes along with keratin flakes that confirmed the diagnosis of dermoid cyst.

A pus culture showed no growth after 48 hours of incubation at 37°C.

After one month of follow-up, there was no abnormal neurological sequelae, the improvement in motor power was grade 4/5 in both lower limbs and there was restoration of light touch and pinprick sensation. The patient was able to mobilize and walk with support.

## Discussion

Intraspinal dermoid cysts, also termed ‘spinal cutaneous inclusion tumors’ are rare, benign, slow-growing tumors that account for less than 1 percent of spinal tumors [8] and arise due to improper separation of the neuroectoderm from the surface ectoderm.

Inclusion dermoids are characterized either into congenital or implanted arrays, the former being more frequent as well as important. A frequent cause of development of such cysts is assertive admittance of the ectodermal cells subsequent to an injury [9]. The patient in this case denied any such history. The cyst presentation at different spinal levels shows a discrepancy. Two of the cases reported from India [2,3] revealed an intramedullary cyst at levels L4-S2 and C3-D2 respectively.

In the lumbosacral region, epidermoid tumor is more common, dermoid tumor is relatively uncommon and the intramedullary manifestation of a dermoid tumor is an exceptionally rare occurrence [10,11].

They remain clinically silent until symptoms appear in the second or third decade of life [12]. Muthukumar *et al*. presented a case report that corresponded with the MRI findings, however, our case is atypical with regard to the absence of bladder symptoms [2]. Moreover, Shah *et al*. reported the case of a 3-year-old boy, indicating that symptoms may develop earlier in some cases [4].

These cysts are often diagnosed when presenting clinically as neurologic manifestations, ranging from paresthesias to paralysis and sphincter complications [13]. In this case, the patient presented with numbness, weakness and paresis progressing to complete immobility. The case series of epidermoid and dermoid tumors of the spinal cord presented by Bradford evidenced most of the cases with urinary symptoms, either retention or incontinence [11], however, the patient in our case denied any of these symptoms but rather presented with bowel disturbance. Gercek *et al.* also reported the case of a patient with a spinal dermoid cyst with a dissynergic bladder [14].

This case gives an excellent clinical picture of a spinal intramedullary dermoid cyst without the classic symptoms of incontinence or signs of spinal dysraphisms.

Our case report also calls attention to the diagnosis of dermoid cyst in spinal surgical patients with the help of MRI and histopathological evaluations. Our patient was initially diagnosed as having TB of the spine due to conventional conjecture and overlapping symptoms. In the case of misdiagnosis, the rupture of the cyst can result in arachnoid, meningeal or cerebral irritation as a result of dissemination through cerebrospinal fluid pathways [12].

The pus culture did not reveal any bacterial growth (including acid-fast bacilli (AFB)) and further excluded the diagnosis of TB of the spine.

The treatment of choice for the dermoid tumor is the total excision of the mass at an early stage. Total mass excision is possible for extramedullary dermoid cysts; however, in intramedullary dermoid cysts, the capsule adheres to the cord [13] and often leads to difficulties in their complete resection through surgery [15]. In our case, the tumor had well-demarcated boundaries hence total resection was accomplished.

## Conclusions

In conclusion, our case is unusual in relation to the absence of the classic features of dermoid cysts and with the atypical symptoms of fecal retention.

The patient presented to us six months after the initial complaint, with a misdiagnosis of spinal TB. Such a delay can progressively worsen the condition. Therefore, these cases need appropriate diagnosis, which should entail the critical evaluation of every case, and for histopathology and radiological examinations to be conducted to ensure the well-being of the patients. It is extremely important to be cautious during surgical resection of such tumors, as a rupture can lead to severe consequences.

## Consent

Written informed consent was obtained from the patient’s legal guardian for publication of this manuscript and accompanying images. A copy of the written consent is available for review by the Editor-in-Chief of this journal.

## Abbreviations

AFB: Acid-fast bacilli; ATT: Antituberculous therapy; MRI: Magnetic resonance imaging; RMO: Registered medical officer; TB: Tuberculosis

## Competing interests

The authors declare that they have no competing interests.

## Authors’ contributions

MS conceived the idea, reviewed and wrote the manuscript. SM and AA contributed to writing the manuscript. ASMH performed the surgery on the patient. SB critically reviewed the manuscript. All authors read and approved the final manuscript.
